# Longitudinal Changes in Hearing Aid Use and Hearing Aid Management Challenges in Infants

**DOI:** 10.1097/AUD.0000000000000986

**Published:** 2020-12-23

**Authors:** Anisa Sadru Visram, Amber Jemima Roughley, Caroline Louise Hudson, Suzanne Carolyn Purdy, Kevin James Munro

**Affiliations:** 1 University of Manchester NHS Foundation Trust, Manchester Academic Health Science Centre, United Kingdom; 2 Manchester Centre for Audiology and Deafness, School of Health Sciences, University of Manchester, United Kingdom; 3 School of Psychology, University of Auckland, Auckland, New Zealand.

**Keywords:** Hearing aid management, Hearing loss, Infant, Parent challenges, Pediatric audiology

## Abstract

If the benefits of newborn hearing screening and early intervention are to be fully realized, there is a need to understand the challenges of hearing aid management in infants. The aim was to investigate longitudinal changes in hearing aid use and hearing aid management challenges in very young infants.

**Design:**

Eighty-one primarily female (99%) caregivers of infant hearing aid users completed a questionnaire about hearing aid management experiences, first when their infants were 3 to 7 months old (1 to 6 months after hearing aid fitting) and again at 7 to 21 months of age. Hearing aid data logging was compared with caregiver reports of daily use for 66 infants.

**Results:**

The main hearing aid management challenges reported by caregivers were performing listening checks and troubleshooting. These challenges reduced over the approximately 5-month time period but remained a problem with around a quarter of respondents still not confident or unsure about troubleshooting, and around a third not performing a daily listening check. Mean daily hearing aid use, obtained from data logging, declined significantly over time from 6.6 to 5.3 hours. Further analysis revealed reduced hearing aid use was primarily among infants with profound losses (n = 11). Caregivers overestimated daily hours of use at both time points. Caregivers reported difficulty with the infants pulling out their hearing aids, especially at the later time point.

**Conclusions:**

The findings from this relatively large sample of caregivers of young infants, assessed at two time points, revealed significant challenges in hearing aid management, including highly variable daily hearing aid use. Interventions that use behavior change techniques may be needed to ensure intentions are consistently turned into successful actions, if the benefits of newborn hearing screening and early intervention are to be fully realized.

## Abstract 2

Supplemental Digital Content is available in the text.

## INTRODUCTION

The importance of early and effective intervention for children with hearing loss is well established. In England, a national newborn hearing screen has been in operation since 2006. Since the introduction of the screening program, the median age of first hearing aid fitting has reduced from around 26 months to just 2.7 months (Wood et al. 2015). Early intervention, with hearing aids or cochlear implants, is associated with better outcomes for speech and language and better longer-term educational outcomes such as reading comprehension (Yoshinaga-Itano et al. 1998; Tomblin et al. 2015; Pimperton et al. 2016; Ching et al. 2018a). Higher daily hearing aid use and greater access to language input are associated with better language outcomes (Tomblin et al. 2015; Nittrouer et al. 2020). Tomblin et al. (2015) found that children who wore their hearing aids for over 10 hours/day (according to caregiver reports) showed greater improvement in language scores, between the ages of 2 to 6 years, than those who wore aids for less than 10 hours/day.

The benefits of early intervention and consistent hearing aid use are well established (e.g., Ching et al. 2018a). However, the challenges of effective hearing aid use and management at a young age should not be underestimated (Muñoz et al. 2015). When an infant is diagnosed with a hearing loss, caregivers must receive and understand a lot of information about the hearing loss itself, about hearing aids and how to manage them, and about the support services available. It is understandable that caregivers may be overwhelmed by this information and the need to manage hearing aids effectively, and this has the potential to have a negative impact on effective and consistent hearing aid use. Identifying aspects of hearing aid management that caregivers report as challenging would allow hearing professionals to focus on addressing and supporting these areas, which in turn should increase hearing aid use with the potential to lead to better outcomes.

Caregiver questionnaires have been used to investigate the challenges faced by caregivers of children and infant hearing aid users. Muñoz et al. (2015) used the Parent Hearing Aid Management Inventory (PHAMI) to investigate hearing aid management challenges faced by caregivers recruited from early intervention services from two US states. Caregivers of 37 children, of mean age 22 months, took part. The PHAMI asks caregivers to rate how confident they feel on a number of different hearing aid management skills and to identify factors that influence hearing aid use. It also includes questions about how caregivers feel about the way in which they received information about hearing loss and hearing aids. Although 40% of caregivers who responded said they were overwhelmed by the amount of information they received, most of the caregivers (84%) wanted all of the information as soon as the hearing loss was confirmed and hearing aids first fitted (Muñoz et al. 2015). Over half (54%) of caregivers did recall being taught how to do a listening check on the hearing aids, but only 36% reported that they actually carried out a listening check daily (Muñoz et al. 2015). There were some aspects of hearing aid management that some caregivers did not recall being taught. For example, 32% of the caregivers reported that they were not taught how to troubleshoot faulty hearing aids (Muñoz et al. 2015). Over half of caregivers (53%) were confused about how to make sure the hearing aids stay in throughout the day (Muñoz et al. 2015). Muñoz et al. (2019) used surveys to explore experiences of parents of 0- to 6-year-old hearing device users, sampled from around the world using an online approach. Some issues highlighted included: few parents recalling discussions of hours of use via data logging with their audiologists and only around half receiving “loaner” devices when theirs were being repaired. Most parents performed regular hearing aid checks for wax blockage, physical condition, and batteries, but many reported poor confidence and adherence to carrying out listening checks. Some parents reported challenges of not knowing if the devices were working or if they were providing benefit. Caballero et al. (2017) used surveys with Hispanic US caregivers of child hearing aid users aged 0 to 5 years, finding many wanted more information about peer support and hearing aid maintenance, despite most already having been given the information. Caregivers feared losing the hearing aids, and not seeing benefit from their use. Results from surveys show that caregivers did not feel confident with some aspects of hearing aid management, and this may have prevented them from carrying out routine care such as troubleshooting and listening checks. Also, caregivers may simply not recall some of the hearing aid management information given to them and/or may not have the opportunities or capabilities to put the information into practice, despite being motivated to do so. Training caregivers using a behavior change approach may help address these issues.

Caregivers may lack the required knowledge for good hearing aid management, or may struggle to put this knowledge into practice. In a survey of 349 US audiologists, nearly half (44%) reported they did not routinely teach caregivers all aspects of hearing aid maintenance (e.g., changing microphone covers) and 38% did not routinely show caregivers how to teach other caregivers about hearing aid management (Meibos et al. 2016). Even when caregivers do have the required knowledge and skills for good hearing aid management, it is well known that patients/caregivers do not always adhere to treatments which have been recommended, regardless of their intentions to do so. For example, a meta-analysis of 20 studies investigating adherence to medication for coronary heart disease estimated that adherence was around 57% (Naderi et al. 2012). Greening et al. (2007) reported on parental challenges in adherence to daily treatment tasks in behaviorally problematic children with type I diabetes, and how this could be mediated by instilling routines. A Cochrane review found that studies aiming to increase adherence to treatments for chronic conditions had mixed outcomes, and there was no recommendation for any one type of intervention to increase adherence (Nieuwlaat et al. 2014).

Caregivers may be highly motivated to ensure their babies are wearing functioning hearing aids, but may require specific strategies to develop capabilities and provide opportunities for good hearing aid management and maintenance skills. Behavior change approaches based on the Capability-Opportunity-Motivation-Behavior (COM-B) model have been shown to be potentially useful in adult audiology (Barker et al. 2016; Ekberg et al. 2020). The COM-B model identifies three “sources of behavior” (“capability,” “opportunity,” and “motivation”) around which a “behavior change wheel” has been developed (Michie et al. 2011). This approach has been successfully used in the creation of supplementary online learning material to increase self-efficacy in adult hearing aid users and to investigate how smartphone-connected listening devices might be useful for adults with a hearing loss (Maidment et al. 2019, 2020). Sindrey et al. (2020) used the approach to design video interventions to help families maximize infant hearing aid use, demonstrating one way in which a behavior change approach could be applied to caregivers’ management of their children’s hearing aids.

The children in Muñoz et al.’s (2015) study had a mean age of 22 months, and other questionnaire studies have focused on children aged up to 5 to 6 years (Caballero et al. 2017; Muñoz et al. 2019). If the benefits of early intervention are to be fully realized, there is a need to understand the challenges of hearing aid management from a younger age. This will help inform behavior change techniques (BCTs) to improve hearing aid use and maintenance from when the hearing aids are first prescribed and fitted. Therefore, the aims of this study were to identify which aspects of hearing aid management caregivers of young babies find challenging, how this changes over time, and to investigate hearing aid use at different time points.

## MATERIALS AND METHODS

### Participants

Caregivers of 103 infants with a hearing loss ranging from mild to profound were recruited from 53 NHS audiology departments across the United Kingdom, between June 2016 and February 2019. The families were recruited as part of a larger longitudinal study investigating the feasibility of using aided evoked potential measures to assess infant hearing. Families received an information pack from their audiologist or teacher of the deaf, and contacted the researchers directly if they were interested in taking part. Ethical approval was obtained from the North West National Research Ethics Service Ethics Committee (reference 172044), and all caregivers gave written informed consent to participate. Caregivers completed a hearing aid management questionnaire and data logging was recorded at two time points, which were opportunistic to coincide with participation in the wider study.

Of 103 participants, 17 were excluded from questionnaire analysis due to not fully completing the questionnaire at both time points (i.e., missing answers to more than two non–free-text questions), and five were excluded due to a different caregiver completing the questionnaire at each time point, leaving a total of 81 participants. Eighty of these 81 respondents were female primary caregivers. These 81 infants had a median age of 5.1 months (range 3.4 to 7.3) at the first time point and median age 10.2 months (range 7.4 to 21.4) at the second time point. For premature infants, all ages are reported corrected. The median age at hearing aid fitting was 1.8 months (range 0.6 to 5.0 months), and the median duration of hearing aid use at the initial time point was 3.4 months (range 0.8 to 6.3 months).

Thirty-three of the 103 infants were excluded from data logging analysis due to data logging being unavailable in at least one of the two sessions. A further three were excluded because the data logging appeared unreliable (i.e., >12 hours use with caregiver reports suggesting aids being left switched on when not in use) and a further one was excluded due to not having provided a subjective response about hearing aid use. One case was included in the analysis with an average daily usage of 16.9 hours as the caregiver indicated that hearing aids were worn all day, including for naps. This left 66 cases for data logging analysis (median age 5.1 months (range 3.0 to 7.5) and 10.1 months (range 7.4 to 21.4) at the two time points). Twelve participants included in data logging analysis were not included in the questionnaire analysis.

### Materials

An adapted version of Muñoz et al.’s (2015) PHAMI was used (see the Appendix in Supplemental Digital Content 1, http://links.lww.com/EANDH/A738), whereby questions were removed if they were not relevant to the aims of the study. For example, questions on affordability of batteries and ear moulds were removed as these are free at the point of delivery in the UK National Health Service. The resulting questionnaire covered three main areas: (1) Information provided by the audiology clinic (four Likert-scale questions); (2) hearing aid management skills (12 Likert-scale questions); and (3) factors influencing hearing aid use (nine Likert-scale questions). Likert-scale questionnaire responses were given a numeric value from 1 (strongly agree) to 5 (strongly disagree). There were four additional free-text questions for caregivers to provide further details about the three main areas and any other comments they had on their experience of their child’s hearing aid use. Caregivers were also asked how often their child wore their hearing aids: *all waking hours, most of the day (over 5 hours), some of the day (1 to 5 hours), or hardly at all (less than 1 hour).*

### Procedures

Caregivers completed a paper version of the questionnaire at both time points. The visits at each time point were arranged as part of the wider research study and were not part of the participants’ regular clinical care. Most caregivers completed the questionnaire on the day they were seen for the main study, and were given at least 1 hour to complete the questionnaire. Participants were able to ask the research audiologist for clarification on the questions as needed. A small number of participants completed it over the following few days and returned the questionnaire by post. Data logging was recorded from hearing aids at both time points, to give an objective measure of daily hours of hearing aid use. Data logging was not typically shared with the caregivers, but was discussed in some cases. Where two aids were worn, data logging was taken as the mean for the two aids.

### Analysis

Where paired-samples *t*-tests were performed, normal distribution of the difference scores was confirmed visually using histograms and box plots. Values from the Likert-scale questions were used to determine mean questionnaire scores. Mean differences between the two time points were analyzed using paired-sample *t*-tests, excluding cases where no response was given, or the response was “not applicable.” To determine significant differences between time points in questionnaire responses (n = 81), corrections were made for multiple comparisons based on the number of questions within each category of questions (i.e., in the first section with four questions, a corrected significance threshold of 0.05/4 = 0.0125 was used; in the second section with 12 questions, 0.05/12 = 0.0042 was used; in the third section with nine questions, 0.05/9 = 0.0056 was used).

For the four free-text questions a qualitative content analysis (Vaismoradi et al. 2013) was initially performed by the second author and reviewed by the first author. An inductive approach was used to code the data and derive categories directly from the data (Cho & Lee 2014). Responses from all participants (including those with otherwise incomplete questionnaires) were included in the content analysis (n = 99 early time point, n = 92 later time point).

Differences in data logging between the two time points were compared using a paired-samples *t*-test. To compare data logging with caregiver reported use, data logging values were converted into categorical data (<1, 1 to 5, and >5 hours) and compared with the equivalent categories used in the caregiver-reported data. The categorical data were assigned a numerical value (1 to 3), and agreement between categorical scores for subjective (caregiver) and objective (data logging) values was determined using paired-samples *t*-tests for the n = 66 participants with both data logging and caregiver report available.

## RESULTS

### Questionnaire Section 1: Information About Hearing Loss and Hearing Aids Provided by Audiology Clinic

**Fig. 1.**
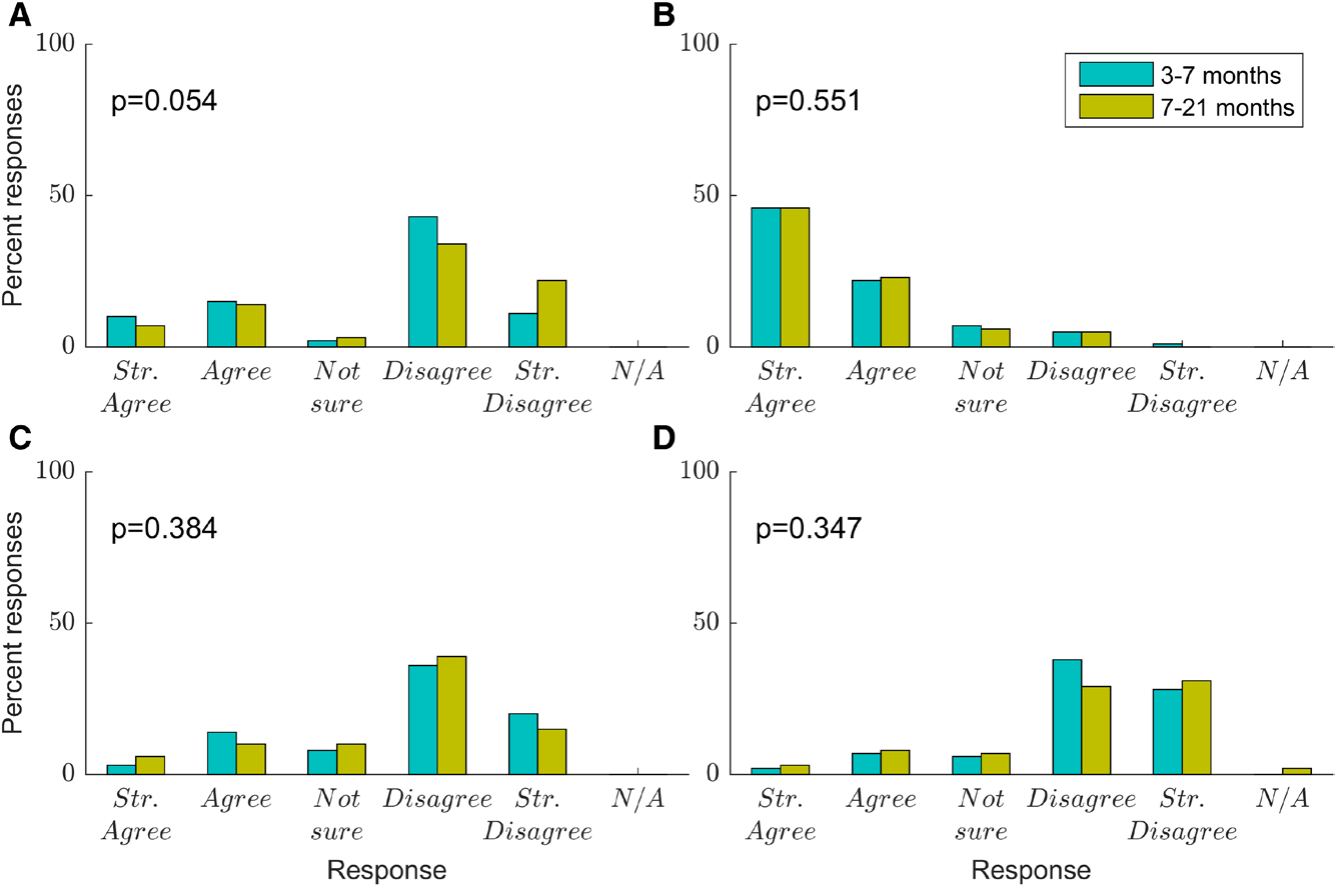
Caregiver responses to questions relating to information about hearing loss and hearing aids provided by audiology clinic (blue, 3–7 months; mustard, 7–21 months). *p* values are shown for paired-samples t-tests comparing scores at the two time points. The significance threshold was taken as *p* < 0.013, to correct for four comparisons (n = 81). A, I was overwhelmed by the amount of information I was given. B, I wanted to have all the information on hand from the beginning. C, I wanted to get information gradually. D, I was not given enough information, I wanted more details.

Figure 1A shows that while the majority of caregivers did not report feeling overwhelmed by the amount of information given to them by the audiology clinic, around a quarter to a third (31% at the early time point and 26% at the later time point) of caregivers did feel overwhelmed. On average, caregivers reported feeling less overwhelmed at the later time point, but this effect did not reach significance (*t*(79) = 1.96, *p* = 0.054). Figure 1B shows caregivers tended to agree or strongly agree that they wanted all the information from the beginning (85%), and this did not change over time. Figure 1C shows whether caregivers wanted to receive information gradually (a similar but differently framed question to Fig. 1B). The majority reported that they did not want to receive information gradually (68%), but some did (20%) or were not sure (11%). This did not change significantly over time. Figure 1D shows most caregivers felt they were given enough details: only 11% at the early time point and 14% at the later time point felt they wanted more details, which did not represent a significant change over time.

### Information About Hearing Loss and Hearing Aids Provided by Audiology Clinic: Free Text

The response categories emerging from the question “What other information would have been helpful?” are summarized in Table 1, ranked in order of occurrence. The main categories were: what their infant could and could not hear with and without the hearing aids (e.g., “the only struggle was not knowing what she could and could not hear, this was not made clear to me”); what the ongoing care plan would be (e.g., “A rough plan of action on paper in regards to further tests”); access to peer support (e.g., “…what we really needed in those first few weeks post diagnosis was to meet a local family in a similar situation that could show us it is not as scary as it first seems”); and what caregivers could expect about their infant’s future (e.g., “information for the future [school etc]”). Caregivers also highlighted aspects of hearing aid management; this topic was addressed further in the following section of the questionnaire.

**TABLE 1.** Categories identified from answers to the free-text question: “What other information would have been helpful?”

Category Identified	Response Frequency (%)		
	Overall (n = 56)	3–7 mo (n = 45)	7–21 mo (n = 28)
Hearing aid management	23	18	21
What infant can hear with/without aids	20	16	18
Plan in audiology going forward (further appointments, etc.)	14	13	7
Peer support	13	7	18
What to expect for infant in the future	13	16	0
More information about hearing tests	7	7	4
British sign language information	5	4	4
Written information	4	4	0
Online resources	4	0	7
Repetition of information	4	0	7
Coping with shock of news of diagnosis	3	2	1
Dealing with others’ perceptions	2	2	0
Lack of prompt information on need for lockable battery drawer	2	0	2

Response frequency indicates the percentage of caregivers who referred to the given category, out of all those who replied to this question (given by “n”).

published online ahead of print December 23, 2020.

### Questionnaire Section 2: Hearing Aid Management Skills

**Fig. 2.**
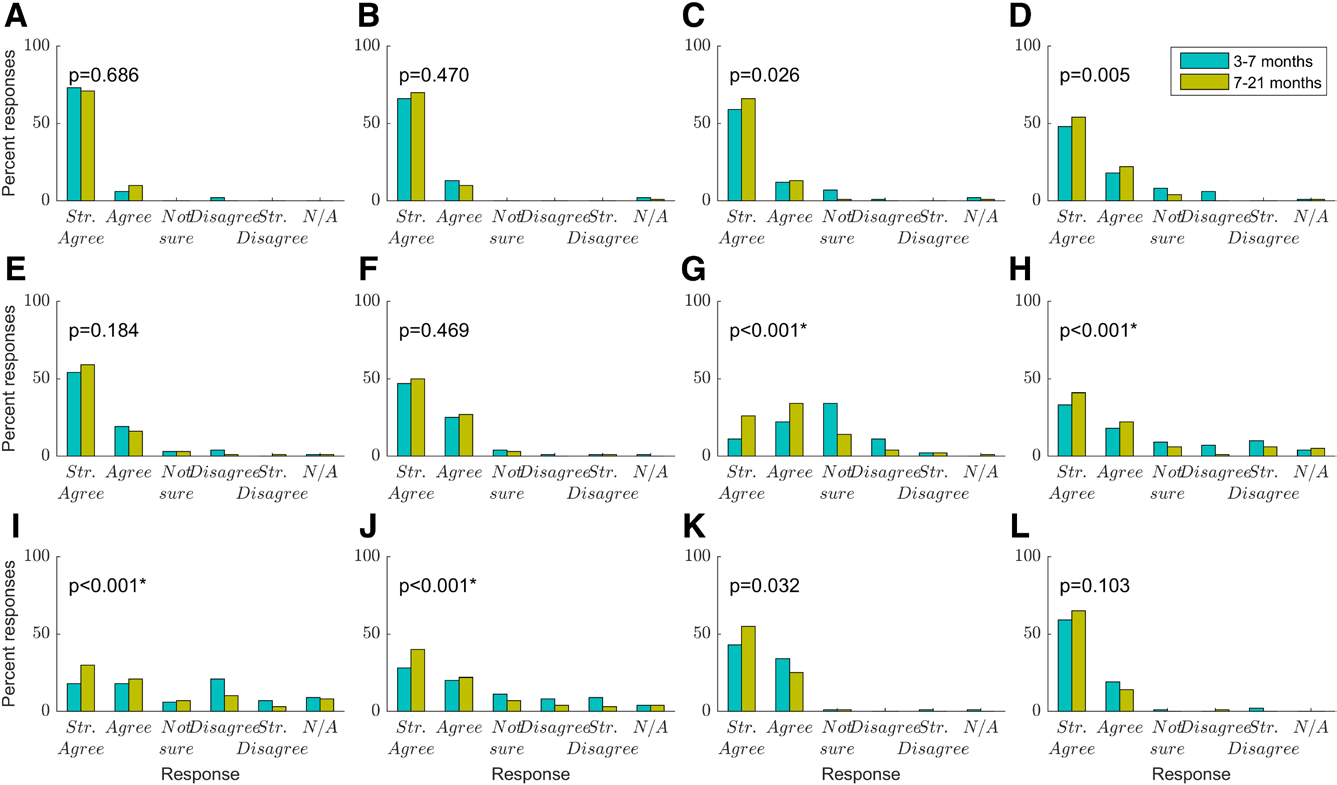
Caregiver responses to questions relating to hearing aid management skills (blue, 3–7 months; mustard, 7–21 months). *p* values are shown for paired-samples t-tests comparing scores at the two time points. The significance threshold was taken as *p* < 0.004, to correct for 12 comparisons. Significant differences are shown with an asterisk (n = 81). A, I am confident in changing hearing aid batteries. B, I am confident in inserting earmolds. C, I am confident in cleaning earmolds. D, I am confident in telling when new earmolds are needed. E, I can reattach the earmold tubing to the hearing aid. F, I know how to try to keep hearing aids on my child. G, I know how to troubleshoot problems with my child’s hearing aids. H, I know how to do a daily listening check with the listening stethoscope. I, If so, I perform a daily listening check. J, I can teach others how to do a listening check of the hearing aids. K, I can teach others how to put the child’s hearing aids on. L, I am confident to emphasize to others the importance of keeping the hearing aids on my child.

Figure 2A–C shows that caregivers reported being highly confident at changing hearing aid batteries, inserting earmolds, and cleaning earmolds, with most “strongly agreeing” they were confident in these skills, and almost all agreeing to some extent. This did not change significantly between the two time points. Figure 2D shows most caregivers agreed they knew when new earmolds were needed, though at the earlier time point a few disagreed or were not sure. Reported confidence in this skill increased over time, reaching borderline significance when corrected for multiple comparisons (*t*(79) = 2.87, *p* = 0.005). Figures 2E and F show that most caregivers strongly agreed, and almost all at least agreed, that they were confident in reattaching earmold tubing and knowing how to keep hearing aids on their child, respectively. This did not change over time. Figure 2G shows a wide spread of responses as to whether caregivers knew how to troubleshoot problems with their child’s hearing aids. At the earlier time point, only 41% agreed they were confident, whilst 16% disagreed, and 43% were not sure. At the later time point, confidence increased with 74% agreeing they were confident, 7% disagreeing, and 17% not sure. This increase in confidence over time was significant (*t*(79) = 5.51, *p* < 0.001). Figure 2H shows a majority of caregivers agreed they knew how to perform a daily listening check (63% at the early time point and 78% at the later time point), but with some spread across the categories (21% vs. 9% disagreed at the early and late time points respectively; 11% vs. 7% reported being unsure). The increase over time in reports of knowing how to perform a listening check was significant (*t*(76) = 4.06, *p* < 0.001). Figure 2I shows a wide spread of responses as to whether a daily listening check was actually performed, with significantly more agreement from caregivers that they performed such a check at the later time point (*t*(67) = 5.08, *p* < 0.001). Figure 2J shows a similar response pattern for caregiver confidence in teaching others how to do a daily listening check and knowing how to do the listening check themselves, with most caregivers agreeing, and confidence significantly increasing over time (*t*(74) = 4.45, *p* < 0.001). Figures 2K and L show caregivers agreed that they were confident in teaching others how to put the child’s hearing aids on (98%) and in emphasizing to others the importance of keeping hearing aids on their child (98%). These reports did not vary significantly over time.

### Hearing Aid Management Skills: Free Text

Response categories emerging from the question: “What other support would have been helpful in learning hearing aid management?” are summarized in Table 2. The dominant category from this question, when answered at the first time point (3 to 7 months), was a desire for more information about how to perform a daily listening check. Many caregivers did not know how to do a listening check, did not know that they should do so, and/or did not have the tools to do so (e.g., “I have not been shown how to perform a listening check which would be useful”; “I did not realize to check them daily”; “what is a listening stethoscope?”). This was no longer a frequent comment at the later time point (7 to 21 months), suggesting that caregivers had been spurred to educate themselves (or had received support to learn) on this topic, and presumably had discussed it with their hearing professionals. At the early time point, 30% of caregivers who responded to the question reported listening checks as an area in which they required support. By the second time point, no caregivers highlighted this as an issue. Another common response at the early time point was a desire for more information about cleaning the hearing aids (e.g., “Reminder of how to clean hearing aids and to have the recommended frequency for cleaning spelt out”). Support with inserting and keeping moulds in the ears was commented on at both time points (e.g., “all the options to keep hearing aids on from stickers to headbands”). Support for retubing was a response that occurred more frequently at the later time point (e.g., “practicing retubing with someone watching”).

**TABLE 2.** Categories identified from answers to the free-text question: “What other support would have been helpful in learning hearing aid management?”

Category Identified	Response Frequency (%)		
	Overall (n = 51)	3–7 mo (n = 44)	7–21 mo (n = 23)
Listening checks	25	30	0
Cleaning	20	20	4
Information about putting/keeping moulds in	18	11	22
Retubing	14	5	22
Moisture management	10	7	9
Information for relatives/other caregivers	8	7	9
Courses/classes	8	5	13
More accessories	6	5	4
Other sources of information available	6	7	0
Checking/changing batteries	4	5	0
Written information packs	2	0	4

Response frequency indicates the percentage of caregivers who referred to the given category, out of all those who replied to this question (given by “n”).

### Questionnaire Section 3: Factors Having a Negative Impact on Hearing Aid Use

**Fig. 3.**
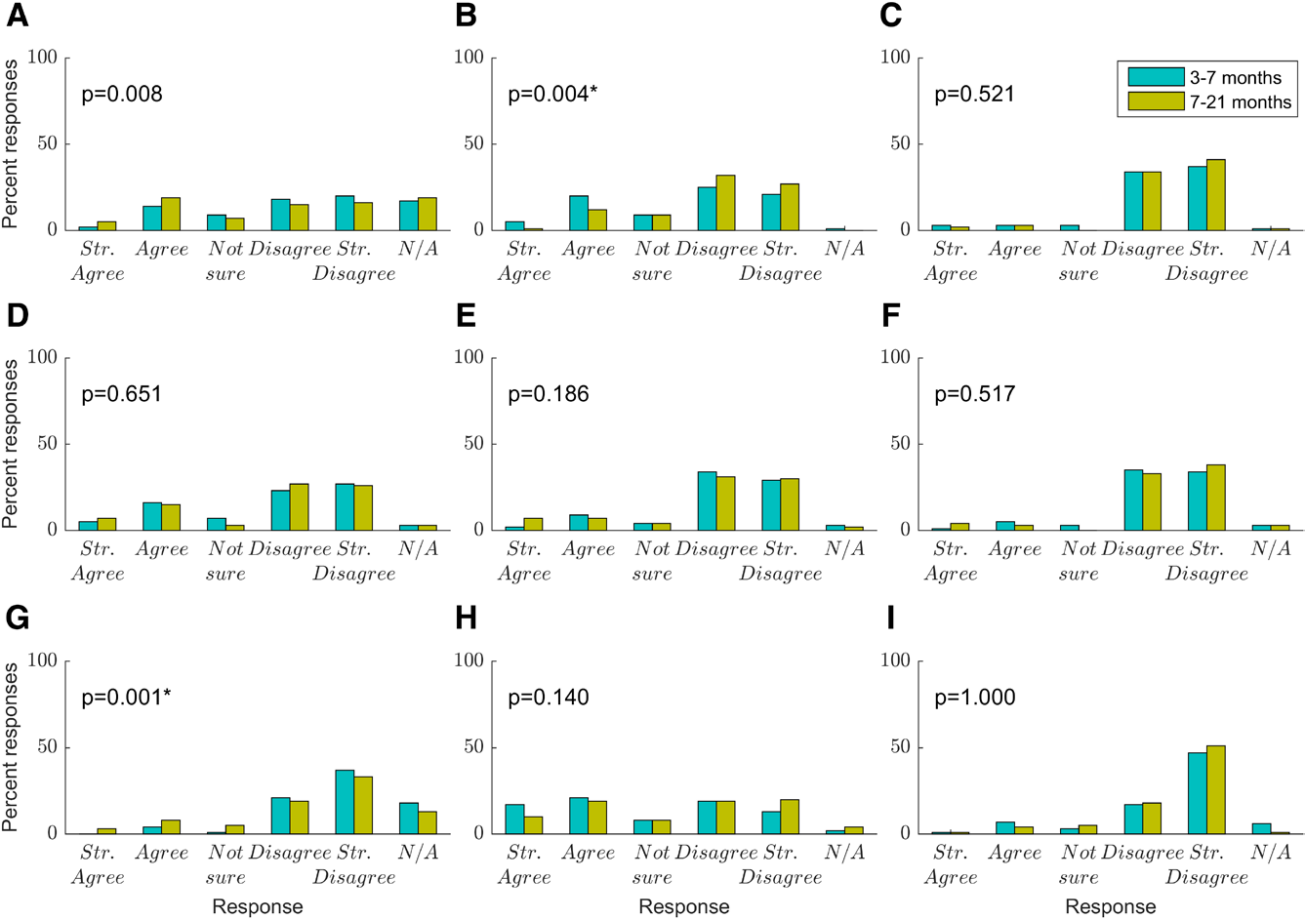
Factors affecting hearing aid use. These questions were prefixed with the statement: “The following factors have a negative impact on my child’s hearing aid use”: (blue, 3–7 months; mustard, 7–21 months). *p* values are shown for paired-samples t-tests comparing scores at the two time points. The significance threshold was taken as *p* < 0.006, to correct for nine comparisons. Significant differences are shown with an asterisk (n = 81). A, Distractions and needs of other children in the home. B, Difficulty getting into a set routine. C, My ability to manage the hearing aids. D, The hearing aids not working correctly. E, A long wait for an appointment with the audiologist. F, A lack of response from the audiologist when I have questions. G, Frequent ear infections. H, Frequent feedback (whistling and squealing) from the hearing aids. I, Insecurities with the appearance of the hearing aids.

Figure 3 shows factors that caregivers reported as having a negative impact on their child’s hearing aid use. Figure 3A shows a wide range of responses as to whether caregivers agreed that distractions and needs of other children in the home had a negative impact on their child’s hearing aid use. More caregivers reported this was an issue at the later time point (30%) than at the early time point (20%). This effect approached but did not reach significance after correction for multiple comparisons (*t*(62) = 2.73, *p* = 0.008). Figure 3B shows that a majority of caregivers disagreed that difficulty getting into a routine had an impact on their child’s hearing aid use, but many did agree that this had an impact (31% at the early time point and 16% at the later time point). On average, caregivers reported this was less of an issue at the later time point, and this difference was statistically significant (*t*(79) = 3.01, *p* = 0.004). Figure 3C shows that most caregivers (88% at the early time point and 93% at the later time point) reported their ability to manage the hearing aids did not have a negative impact on use, and this did not change significantly over time. Figure 3D shows a range of responses as to whether “hearing aids not working correctly” impacted use, with more disagreeing (64%) than agreeing (27%) and no significant change over time. Figure 3E shows most caregivers did not find a long wait for an audiologist appointment was an issue, but for a small number this was a factor (14% at the initial time point and 17% at the later time point). There was no significant change over time. Figure 3F shows that the majority of caregivers (86%) did not report problems with audiologists not answering their questions, while only 8% did report this as a problem. There was no significant change over time. Figure 3G shows that frequent ear infections were an issue for only a small number of caregivers, increasingly at the later time point (5% and 14% at the early and later time points). This increase over time was significant (*t*(62) = 3.40, *p* = 0.001). Figure 3H shows a very wide range of responses across all categories as to whether frequent feedback impacted hearing aid use, with no significant change over time. Figure 3I shows most caregivers strongly disagreed that insecurities with the appearance of the hearing aids impacted their use, and this did not change over time.

### Factors Having a Negative Impact on Hearing Aid Use: Free Text

Table 3 summarizes the categories of responses emerging in answer to the prompt: “Any other reasons that make my child’s hearing aid use difficult.” By far, the most common response was difficulty with the infant removing the hearing aids from their ears (e.g., “Constantly pulling out hearing aids and putting them in his mouth”). This occurred frequently at both time points, but even more so at the older age (36% of those who responded to the question highlighted aids being pulled out at the early time point, and 65% did at the later time point). The next most common categories were poorly fitting earmolds (e.g., “when the moulds are getting small they fall off I do not notice at first or have not got time to replace them”), and earmolds taking too long to arrive (e.g., “baby grows so quickly means needing new moulds regularly and the time taken to receive them back means being unable to use the aids as much as we should be”). Both areas of difficulty were more frequently mentioned at the earlier time point.

**TABLE 3.** Categories identified from answers to the free-text prompt: “Any other reasons that make my child’s hearing aid use difficult”

Category Identified	Response Frequency (%)		
	Overall (n = 73)	3–7 mo (n = 58)	7–21 mo (n = 57)
Infant removing hearing aids	62	36	65
Poorly fitting ear moulds	22	24	5
New ear moulds taking too long	15	12	9
Car journeys	10	5	9
Feedback	8	7	4
Noisy environments	8	5	7
Infant dislikes aids	7	9	2
Water	7	5	5
Infections/skin problems/wax	5	7	0
Other caregivers not maintaining use	1	2	0

Response frequency indicates the percentage of caregivers who referred to the given category, out of all those who replied to this question (given by “n”).

### Other Comments: Free Text

A few new categories emerged from the final prompt: “Any other comments about my experiences with my child’s hearing aids.” A large number of caregivers reported positive overall experiences with audiology services and other professionals (e.g., “Overall support from audiology, teachers of the deaf and pediatricians have been FANTASTIC”), and positive experiences of using the hearing aids (“I am grateful for the hearing aids to give my child better hearing to help with development”). Minor categories included: problems attending audiology appointments (e.g., “…constant appointments and ability to attend”; “The only difficulty has been with our distance to ****** Hospital”) and difficulties with public perception/awareness of hearing loss (e.g. “I feel people look at him when he is out”; “It is difficult managing comments about them. Not discrete like some adult aids”).

### Hearing Aid Use: Data Logging and Self-report

**Fig. 4.**
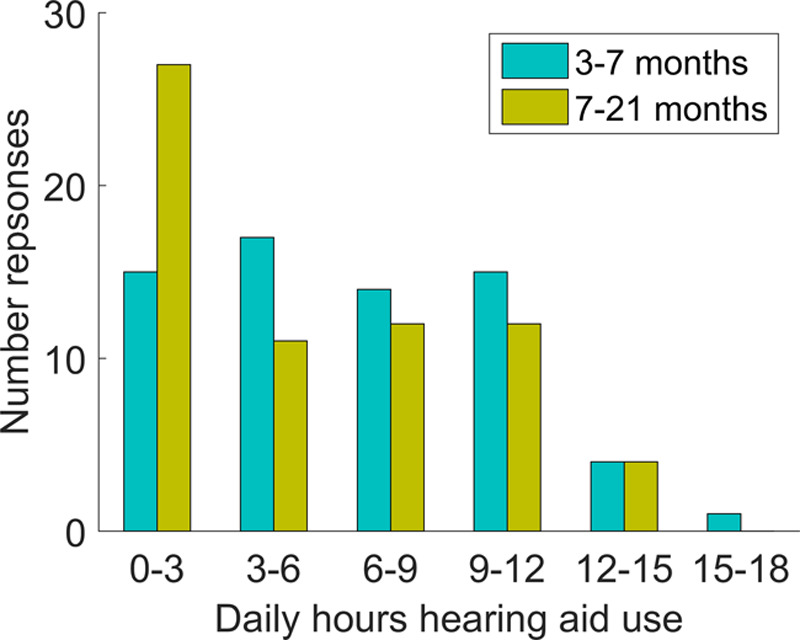
Daily hours’ hearing aid use according to data logging (n = 66).

Figure 4 shows daily hours’ use at both time points according to hearing aid data logging (n = 66). At the early time point, there was a wide range of daily use with mean 6.6 hours (range 0.3 to 16.9). By the second time point, there was still a wide range of daily use times but a greater number were now using their hearing aids less than 3 hours/day. At the older age, mean daily hours’ use was 5.3 hours (range 0 to 13.5). This reduction in daily hours of use at the later time point was statistically significant (*t*(65) = 3.14, *p* = 0.003). There was a statistically significant correlation between reduction in daily hours’ hearing aid use and four-frequency average hearing threshold, with greater reduction in use being associated with greater degrees of hearing loss (*R* = 0.326, *p* = 0.013). Further analysis revealed that for the n = 11 infants with profound loss (better ear four-frequency average hearing threshold > 95 dB HL), mean daily use reduced significantly from 7.7 hours at the early time point to 4.2 hours at the later time point (*t*(10) = 4.90, *p* < 0.001). This remained significant excluding two infants with likely progressive loss between the two time points (*t*(8) = 3.94, *p* = 0.004). For the remaining n = 55 infants without profound loss, there was a trend for a decline in use from 6.4 to 5.6 hours, but this did not reach statistical significance (*t*(54) = 1.87, *p* = 0.067). For cases where bilateral data logging was available (n = 61) the median absolute difference between the left and right data logging was 0.3 hours at the early time point and 0.4 hours at the later time point, indicating largely bilateral hearing aid use for these infants.

**Fig. 5.**
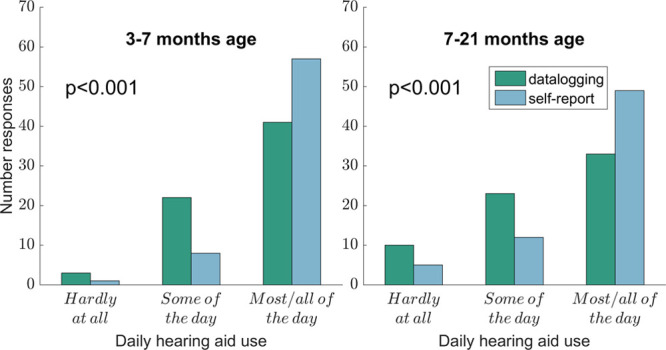
Data logging and caregiver reports of daily hearing aid use at the two time points (n = 66).

Figure 5 shows caregiver reports (n = 66) of daily hearing aid use alongside data logging for comparison, with the two time points in separate panels. The data logging values were converted into equivalent categorical data (“hardly at all” <1 hour; “some of the day” 1 to 5 hours; “most/all of the day” >5 hours). For analysis, the “most of the day” and “all waking hours” categories from caregiver self-reports were combined into a single category. Figure 5 highlights caregivers’ overestimation of daily hearing aid use at both time points. At the first time point, 86% of caregivers (57 of 66) reported their child using the aid(s) for over 5 hours/day, but only 62% (41 of 66) were shown to be used for over 5 hours by data logging. At the second time point, 74% of caregivers (49 of 66) reported their child using the aid(s) for over 5 hours/day, but only 50% (33 of 66) were shown to be used for over 5 hours by data logging.

Paired-samples *t*-tests (based on assigned scores of 1, 2, and 3 to the respective categories) at the two time points revealed that at both time points caregivers reported significantly greater daily use than was revealed by data logging (3 to 7 months: *t*(65) = 4.09, *p* < 0.001; 7 to 21 months: *t*(65) = 4.06, *p* < 0.001).

### Relationship Between Questionnaire Answers and Hearing Aid Use

To investigate whether self-reported hearing aid management skills or factors affecting use (sections 2 and 3 of the questionnaire) were negatively associated with objectively measured hearing aid use, scores from these sections of the questionnaire were summed and correlated with hearing aid data logging, for the 54 participants with complete data logging and questionnaire data. No significant correlations were found (early time point/hearing aid management skills *r* = −0.200, *p* = 0.147; early time point/factors impacting hearing aid use *r* = 0.115, *p* = 0.406; late time point/hearing aid management skills *r* = −0.090, *p* = 0.516; late time point/factors impacting hearing aid use *r* = −0.244, *p* = 0.075).

## DISCUSSION

Challenges with hearing aid management, both soon after hearing aid fitting, and after approximately 5 months of hearing aid use were identified in this relatively large sample of infants. Caregiver responses showed improvements in some aspects of hearing aid management over time. Despite this positive finding, data logging showed a significant decline in hearing aid use over time. Responses from 81 caregivers were included in this study, making it a relatively large sample compared to previous studies. Other important features of this study are the young age when the questionnaire was first administered, and the longitudinal design, with data collected at two time points, mostly within the first year of hearing aid use. Previous questionnaire studies investigating caregiver hearing aid management have targeted a wider age range, approximately 0 to 5 years (Muñoz et al. 2015; Caballero et al. 2017; Muñoz et al. 2019).

### Information From Audiology Clinics About Hearing Aids and Hearing Loss

Overall, the majority of caregivers were happy with the amount of information they received from their audiology clinic. Although a quarter to a third of caregivers indicated that they were overwhelmed by the amount of information they received, the majority agreed that they did want all the information from the beginning. Caregivers questioned by Muñoz et al. (2015) also indicated that they wanted all the information from the beginning; however, more caregivers in Muñoz et al.’s study reported feeling overwhelmed (40% in Muñoz et al. compared with 26 to 31% in this study). A higher percentage of caregivers in Muñoz et al.’s study also reported wanting more information: 24% compared with 11 to 14% in this study. These differences may be due to different practices in the United States and the United Kingdom, and perhaps were also affected by infants in our study being younger and hence their diagnosis being in more recent memory. It may also be due to the way in which the families were recruited. Participants in the current study were taking part in a wider study involving them volunteering a significant amount of time with no direct benefit as individuals. This may have led to a bias in the current sample towards more highly motivated caregivers, compared to those who only had to complete a survey.

When asked in a free-text question what other information would have been helpful, access to peer support was frequently reported. Less than half the audiologists surveyed in Meibos et al. (2016) reported routinely providing families with peer support information. Although Meibos et al. surveyed US audiologists, it is likely that this practice is similar in the United Kingdom, where appointment times are limited and there is a need to provide a lot of information. In a recent literature review, peer support was highlighted as having a number of benefits for families of children with hearing loss, including caregiver emotional well-being, and being able to share knowledge (Henderson et al. 2014).

Caregivers reported wanting to know more about how much their babies could hear. Some caregivers specifically mentioned the benefits of listening to hearing loss simulators (e.g., “I was shown a Flintstones sketch on YouTube by our hearing support teacher which really helped me understand. This would have been good to be shown this or something similar early on.”). This suggests that such practical demonstrations are helpful to convey this information. Caregivers also wanted to know about their infant’s future. Whilst the ability to predict longer-term outcomes is limited, hearing/educational professionals could perhaps provide more information about educational support services in the early months after diagnosis. Ching et al.’s (2018b) thematic analysis of interviews of parents of preschoolers with hearing impairment highlighted the important contributions of information obtained online and via services, peer support, and professional advice in shaping parents’ decisions about their child’s future. Having peer support from other families of children with hearing loss could also help to lessen caregivers’ anxieties about the future when first diagnosed (Henderson et al. 2014).

### Hearing Aid Management Skills

Caregivers generally reported feeling confident with most aspects of hearing aid management including changing hearing aid batteries (98 to 100%), inserting earmolds (98 to 99%), and cleaning earmolds (88 to 98%). Confidence knowing when new earmolds were needed increased over time, with 82% agreeing they were confident at the early time point and 94% agreeing they were confident at the later time point. As the caregivers taking part in this study are likely to be highly motivated, it is possible that these results overestimate caregivers’ success with hearing aid management; other caregivers may struggle more with hearing aid management tasks. In general, caregivers who are struggling more with their child’s hearing loss and hearing instruments may be less motivated to participate in research.

There are some areas in which caregivers reported lower confidence when the child was younger and, although there was some improvement over time, these remained a challenge. An example of this was daily listening checks using a stethoscope. At the early time point, around a third of caregivers did not agree that they knew how to do a listening check or how to teach others to do a listening check and less than half carried out the check daily. Caregivers felt more confident about listening checks when their infant was older. Listening checks have previously been reported by caregivers as something they do not feel confident doing (Muñoz et al. 2015, 2019). This has also been highlighted as an area that audiologists do not regularly cover with caregivers (Meibos et al. 2016). Although confidence around listening checks increased in this study as the infants got older, around a third of caregivers of infants aged 7 to 21 months were still not carrying them out daily. Muñoz et al. (2015) reported that only 36% of caregivers carried out a daily listening check in their study of children aged 22 months on average. Additional follow-up is needed to see if rates of listening checks will decline over time to this low level in the current sample of caregivers.

Another area that caregivers were unsure about at both time points was troubleshooting, with over half of caregivers answering “unsure” or disagreeing that they had the skills when their infant was 3 to 7 months. While significantly more caregivers reported they knew how to do this when their babies were 7 to 21 months, over a quarter still reported that they were unsure or disagreed. This is consistent with Muñoz et al.’s (2015) finding that caregivers all reported low confidence in this skill.

### Factors Affecting Hearing Aid Use

Two primary areas that caregivers felt were affecting their infant’s hearing aid use, across both age groups, were frequent acoustic feedback and the hearing aids not working correctly. Although a quarter of caregivers reported that hearing aids not working correctly did affect hearing aid use, we do not know to what extent they felt it affected use (e.g., weeks spent without aids vs. a few hours until the problems were resolved). Faulty hearing aids are something that can be easily addressed if caregivers have appropriate skills, clear information, and access to services. These findings suggest that caregivers need better support to be able to troubleshoot the aids themselves, carry out basic maintenance, and know how and when to ask for support to get replacement aids or have the problem corrected professionally.

Feedback is a slightly trickier issue, especially when the infant is very young and growing rapidly out of their earmolds. When asked in a free-text question what other issues affect hearing aid use, poorly fitting moulds, and the time it takes for new moulds to arrive were cited as problems by a number of caregivers. Feedback suppression features on the hearing aids should help to reduce the amount of feedback whilst enabling the hearing aids to continue giving a high level of gain, however this feature can distort sound, so regularly replacing ear moulds to reduce feedback is important (Chung 2004) particularly for those with more high-frequency gain. Digital feedback management is recommended in national guidelines for pediatric hearing aid fitting (e.g., Feirn et al. 2014; Bagatto et al. 2019) where alternative measures such as well-fitting earmoulds are not sufficient to control feedback, but the use of this technology was not documented in the current study. Having a protocol in place locally in which hearing professionals routinely take new impressions in a timely manner so that new moulds are ready just as, or before, an infant grows out of their current ones may be a solution to this. This may be facilitated in the future by 3D scanning ear impression technology.

Another two areas that many caregivers agreed affected their infant’s hearing aid use relate more to family life than to the hearing aids themselves. These included distractions and needs of other children, and difficulty getting into a set routine. When the infants were younger, one-fifth of caregivers reported that the needs of other children affected hearing aid use, and this increased to nearly one third when the infants were 7 to 21 months. Conversely, when the infant was older, and the families had more experience with hearing aids and hearing loss, fewer caregivers (16%) agreed that getting into a routine affected hearing aid use compared to when the infant was younger (31%). These are difficult areas for hearing professionals to address, as family life and the needs of other children will always continue after diagnosis of a hearing loss. Teachers of the Deaf and other early intervention professionals are better placed than audiologists to offer support in these areas as they have more involvement with the family in their home. The process by which Teachers of the Deaf could provide this support and how hearing aid management fits in with caregivers’ existing routines is a topic for future research.

When asked in a free-text question what other reasons make their child’s hearing aid use difficult, a great many caregivers mentioned their child removing their hearing aids. This is despite the majority of caregivers agreeing or strongly agreeing that they knew how to keep the hearing aids on their child. More caregivers mentioned this when their infant was 7 to 21 months compared to 3 to 7 months, although this was by far the most cited difficulty at both time points. This is in keeping with previous studies, such as Muñoz et al. (2015), where over half (53%) of caregivers reported feeling unsure how to keep the hearing aids in. Moeller et al. (2009) also cited concerns about the child removing the hearing aid as a reason for reduced hearing aid use, and caregivers in Moeller et al.’s study reported that the child wore the hearing aids more where they could be closely supervised. Retention devices are an important tool to help parents keep hearing aids on their child, but Anderson and Madell (2014) reported that many parents had limited knowledge of the range of options for retention devices. Anderson and Madell recommended that hearing professionals ensure caregivers are familiar with the full range of devices available, so they can choose the device that best suits their child. This sentiment was echoed in caregivers’ free-text comments.

### Daily Hearing Aid Use

There was a wide range of daily hearing aid use in both age groups (0 to 16.9 and 0 to 13.5 hours), but hearing aid use was generally low compared with the number of hours an infant is expected to be awake. This is in line with previous research showing that most caregivers struggle to achieve consistent hearing aid use during all waking hours until their children are around 28.5 months, largely due to lower use in situations where infants cannot be closely monitored, for example during car journeys (Moeller et al. 2009). Data logging indicated a significant decrease in use between the early time point (mean usage 6.6 hours) and the later time point (mean usage 5.3 hours). Previous studies of early hearing aid use have shown daily use increases over time (e.g., Jones & Launer 2010; Walker et al. 2015); however, this is difficult to compare as the age range examined varies across studies. In the present study, data from caregivers at two time points, both in early infancy, showed a reduction in use over the first few months of life, which has not been clear from previous studies. This reduction may be somewhat associated with increased reports of infants pulling their aids out, a behavior that is likely to vary and need different solutions across the early childhood period. Overall analysis showed that this effect was dominated by infants with profound losses (who would eventually go on to have cochlear implants). This effect may be related to caregivers not seeing any benefit from ongoing hearing aid use, as was reflected in some caregiver comments: (e.g., “My son has no response to sound even with his hearing aids. This makes it difficult to keep them in his ears.”). For infants with less-than-profound losses, there was a trend for decreased use at the later time point, but this did not reach significance. While mean hours of hearing aid use appear low compared with the number of waking hours, the infants in this study wore their aids more than those in an earlier multicenter US study by Walker et al. (2015) which found that infants aged 6 to 24 months wore their aids 4.4 hours/day on average. The median absolute difference between hours of use in both ears was low (0.3 to 0.4 hours) indicating most caregivers were using both aids rather than just one.

An alternative way to record hearing aid usage is by percentage of waking hours, which may be a better way of recording longitudinal changes, as it accounts for developmental changes in sleeping patterns. This is the approach used in the PEACH (Parents’ Evaluation of Aural/oral performance of Children) questionnaire (Ching & Hill 2007), which asks parents to report use as a percentage, implying use during waking hours only. Marnane and Ching (2015) found in a large sample of children, by age child age 3 years, 71% of caregivers reported consistent hearing aid use of >75% of waking hours, with 62% reporting >75% use during waking hours within the first 12 months of receiving amplification. As we have seen, this is likely to include an overestimate of use, due to being based on parental self-report, though it is not clear how accurately parents report on this type of percentage-based measure. We could expect average sleep per day of around 15 hours in our younger group and 12 hours in our older group (Hirshkowitz et al. 2015), that is 9 and 12 waking hours, respectively. This gives an approximate mean hearing aid use as a percentage of waking hours as 73% at the younger age group and 44% in the older group—making the decline appear even more stark, and hence of likely clinical significance even in the nonprofound group in whom the effect did not reach statistical significance. Data logging gives information on how long hearing aids have been switched on and provides an indirect measure of use. Because hearing aids can be left on when they are removed from the child’s ears, these data may need to be interpreted with some caution. Infants in this study were excluded from the analysis if data logging appeared to be unrealistic based on the history from the caregiver (over 12 hours data logging with no caregiver indication as to why the usage was high), but some inaccuracy in the data logging information is still possible. Although there is a lack of research examining links between daily hearing aid usage and outcomes in infants, there is ample evidence that quantity of language input impacts language outcomes in children with hearing loss (Sultana et al. 2019; Hall 2020; Nittrouer et al. 2020).

Caregiver reported hearing aid use was significantly higher than that recorded from data logging at both time points. This is in keeping with other research which has similarly found that caregivers overestimate hearing aid use and indicates caregiver self-report should not be solely relied upon (Walker et al. 2013; 2015). One explanation for this may be caregivers reporting on typically good days of hearing aid use, rather than appropriately estimating an average. Caballero et al. (2017) showed almost twice as many caregivers reported their infants used hearing aids all waking hours on a good day than on a bad day. A second explanation could be data logging chips reporting use only periodically (around every 15 to 60 minutes) rather than continuously and hence incomplete periods not being counted toward the total use (Husstedt et al. 2017; Timmer et al. 2017). This would particularly bias recorded data logging towards lower numbers than true hours of use for those using their devices intermittently throughout the day, as would be expected for an infant taking regular naps.

### Relationship Between Questionnaire Answers and Hearing Aid Use

No correlation was found between hours of daily hearing aid use and self-reported hearing aid management skills or factors having a negative impact on hearing aid use. Muñoz et al. (2015) did find a correlation between parental reported hearing aid use and extent of hearing aid use challenges reported (i.e., factors negatively impacting use). The current study used data logging, as opposed to caregiver report, and a reduced set of questions on factors affecting use (9 vs. 17 items), which may explain this difference. Although this appears to indicate that difficulties with hearing aid management do not affect hearing aid usage, it is more likely that this is a complex issue that has not been captured in our data.

### Behavior Change Techniques

To address the areas of difficulty with hearing aid management and factors affecting hearing aid use identified in this study, a more targeted behavior change approach may be useful. Having a course/class and being pointed towards other sources of information was suggested by the caregivers when asked what else might be useful in learning hearing aid management (e.g., “A short class/session on hearing aid management or one-one session by professionals”; “…we found videos and information online very helpful”). Recent survey data, since the COVID-19 pandemic, has shown keenness from pediatric audiologists and caregivers to engage in, and expand the scope of, remote care practice (Saunders & Roughley 2020). There is also existing evidence for telepractice being used effectively as a tool for coaching parents to supplement face-to-face clinical sessions (Baharav & Reiser 2010; Daczewitz et al. 2020), including use of videos designed specifically with behavior change models in mind to maximize infant hearing aid use (Sindrey et al. 2020). Future work could develop improved remote practice training, using evidence-based BCTs, to support caregivers’ hearing aid management needs. Michie et al. (2013) developed a BCT taxonomy in which they identified 16 groups of BCTs, for example “goals and planning” and “feedback and monitoring.” Some of these BCTs could be applicable to areas identified in this article. For example, data logging and outcome measures could be used for feedback and monitoring (Michie et al. 2013).

The COM-B behavior change model may be particularly useful for this type of intervention. In this model, “capability,” “opportunity,” and “motivation” are the key factors that need to be influenced in order to change behavior (Michie et al. 2011). The behavior change wheel indicates nine ways in which these factors can be influenced: restrictions, education, persuasion, incentivization, coercion, training, enablement, modeling, and environmental restructuring (Michie et al. 2011). The COM-B approach has been used in the past to increase adherence to treatment and has been shown to be of potential use in adult audiology (Barker et al. 2016). Online tools have been created using the COM-B approach to address hearing aid management and psychosocial factors, which may hinder adherence to hearing aid use (Maidment et al. 2020). Future research could use the COM-B approach to positively influence the practice of hearing professionals and caregiver adherence to treatment and instructions. Target outcomes could include, for example, daily hearing aid use and performance of daily listening checks. Such research would go beyond providing caregivers with the necessary knowledge and skill, and begin to address broader barriers to adherence using evidence-based techniques which could be integrated into future clinical practice.

### Limitations

One limitation of the study is that predominantly (99%) female caregivers were sampled. It would be valuable to determine and compare the experiences of both female and male caregivers of infants from a very young age. Future research on behavior change techniques would need to consider the experiences of all caregivers, potentially including extended family in multi-generational homes (Burgess & Muir 2020). The time points at which the data were sampled were chosen opportunistically to coincide with a wider research study, and so the second time point covered a relatively wide age range (7 to 21 months), and we were not able to hone in on issues specific to a very narrow timeframe of hearing aid experience. Caregiver estimation of hearing aid use was obtained in a categorical manner, rather than asking for an estimate of daily hours use, and this limited how the data could be analyzed, for example correlation analyses could not be reliably performed. The questionnaire in use was a reduced version of the PHMI from Muñoz et al. (2015), and certain factors were not recorded, including caregivers’ emotional challenges, depression ratings, and demographic information. Such data could have provided deeper insights into challenges and barriers to consistent use, and would be valuable in developing successful behavior change approaches in the future.

## CONCLUSIONS

This study identified challenges with hearing aid management for caregivers of young infants, both soon after hearing aid fitting, and after some months of hearing aid use. Caregivers reported difficulty with both troubleshooting aids and infants pulling aids out, both of which can impact daily hearing aid use. By better understanding the challenges facing caregivers, hearing professionals can improve the support they can offer. Caregivers were generally positive regarding information provided at the time of hearing aid fitting, but ongoing challenges in hearing aid management and the reduction over time in daily hours of hearing aid use highlight the need for a new approach to ensure that the benefits of early identification of hearing loss are fully realized.

## ACKNOWLEDGMENTS

The authors are grateful to all the families who took part and all the audiologists and teachers of the deaf around the UK who helped to recruit families to the study. Thanks to Jo Brooks and Rhiannon Morgan for contributing to data collection and to Michael Stone for support on the project. Thanks to Karen Muñoz for permission to use and reproduce sections of the PHMI questionnaire.

## Supplementary Material


