# Supplementary material for: Longitudinal Changes in Hearing Aid Use and Hearing Aid Management Challenges in Infants
Source: Ear Hear. 2020 Dec 23;42(4):961–72. doi: 10.1097/AUD.0000000000000986 (PMC8221722; doi:10.1097/AUD.0000000000000986)
Supplement: Supplementary file 1 [file aud-42-0961-s001.pdf]

Participant ID______________ Date issued_______________

Hearing Aid Management Survey

What is your relationship to the child? Mother/father/other, please state________________________

Child's date of birth ____________________________

Today's date _______________________________

Please answer the following questions about your experiences with understanding and managing your child's hearing aid(s) by ticking the relevant boxes.

**Information About Hearing Loss and Hearing Aids Provided by my Audiology Clinic**	Strongly disagree	Disagree	Not sure	Agree	Strongly agree	Not applicable
1. I was overwhelmed by the amount of information I was given						
2. I wanted to have all the information on hand from the beginning						
3. I wanted to get information gradually						
4. I was not given enough information, I wanted more details						

5. What other information would have been helpful? ………

………

Participant ID______________ Date issued_______________

Hearing Aid Management Skills	Strongly disagree	Disagree	Not sure	Agree	Strongly agree	Not applicable
6. I am confident in changing hearing aid batteries						
7. I am confident in inserting earmoulds						
8. I am confident in cleaning earmoulds						
9. I am confident in telling when new earmoulds are needed						
10. I can re-attach the earmould tubing to the hearing aid						
11. I know how to try to keep hearing aids on my child						
12. I know how to troubleshoot problems with my child's hearing aids						
13. I know how to do a daily listening check with the listening stethoscope						
14. If so, I perform a daily listening check						
15. I can teach others how to do a listening check of the hearing aids						
16. I can teach others how to put the child's hearing aids on						
17. I am confident to emphasize to others the importance of keeping the hearing aids on my child						

18. What other support would have been helpful in learning hearing aid management? ………………………………………………………...

………

Participant ID______________ Date issued_______________

The following factors have a negative impact on my child's hearing aid use:	Strongly disagree	Disagree	Not sure	Agree	Strongly agree	Not applicable
19. Distractions and needs of other children in the home						
20. Difficulty getting into a set routine						
21. My ability to manage the hearing aids						
22. The hearing aids not working correctly						
23. A long wait time for an appointment with the audiologist						
24. A lack of response from the audiologist when I have questions						
25. Frequent ear infections						
26. Frequent feedback (whistling and squealing) from the hearing aids						
27. Insecurities with the appearance of the hearing aids						

Hearing Aid Use

Each day my child typically uses his/ her hearing aids: All waking hours................. most of the day (over 5 hours)............ some of the day (1-5 hours)......... Hardly at all (less than 1 hour)............

Are you or another primary caregiver hearing aid users(s) yourselves?..Y/N...

Any other reasons that make my child's hearing aid use difficult...

....

Any other comments about my experiences with my child's hearing aids...

....

....

For independent information and support about childhood hearing loss you can contact the National Deaf Children's Society https://www.ndcs.org.uk/
